# Analysis of the genetic variation in *Mycobacterium tuberculosis *strains by multiple genome alignments

**DOI:** 10.1186/1756-0500-1-110

**Published:** 2008-11-07

**Authors:** Andrés Cubillos-Ruiz, Juan Morales, María Mercedes Zambrano

**Affiliations:** 1 Corporación Corpogen, Carrera 5 # 66A-34, Bogotá, Colombia; 2 Ingenian Software, Carrera 14 # 90-31 OF. 403 Bogotá, Colombia

## Abstract

**Background:**

The recent determination of the complete nucleotide sequence of several *Mycobacterium tuberculosis *(MTB) genomes allows the use of comparative genomics as a tool for dissecting the nature and consequence of genetic variability within this species. The multiple alignment of the genomes of clinical strains (CDC1551, F11, Haarlem and C), along with the genomes of laboratory strains (H37Rv and H37Ra), provides new insights on the mechanisms of adaptation of this bacterium to the human host.

**Findings:**

The genetic variation found in six *M. tuberculosis *strains does not involve significant genomic rearrangements. Most of the variation results from deletion and transposition events preferentially associated with insertion sequences and genes of the PE/PPE family but not with genes implicated in virulence. Using a Perl-based software *islandsanalyser*, which creates a representation of the genetic variation in the genome, we identified differences in the patterns of distribution and frequency of the polymorphisms across the genome. The identification of genes displaying strain-specific polymorphisms and the extrapolation of the number of strain-specific polymorphisms to an unlimited number of genomes indicates that the different strains contain a limited number of unique polymorphisms.

**Conclusion:**

The comparison of multiple genomes demonstrates that the *M. tuberculosis *genome is currently undergoing an active process of gene decay, analogous to the adaptation process of obligate bacterial symbionts. This observation opens new perspectives into the evolution and the understanding of the pathogenesis of this bacterium.

## Background

The genome of *Mycobacterium tuberculosis *(MTB), considered to be mosaic in structure, has been proposed to be the result of ancient horizontal DNA exchange and subsequent clonal expansion [1]. The MTB genome is highly conserved and is considered a "closed pan-genome" due to the bacterium's restricted niche that limits access to the global microbial gene pool [2]. Despite the limited genetic variation, there is a high degree of phenotypic variability among MTB isolates, including differences in clinical outcome and epidemiological behavior that involve host and environmental factors, as well as bacterial determinants [3,4].

The genome sequences of six MTB strains are available and include the laboratory reference H37Rv [5] and avirulent H37Ra strains and clinical isolates. Strain CDC1551 caused an outbreak with extensive transmission of tuberculosis that is related to increased virulence [6]. The F11 strain represents the largest proportion of all isolates from tuberculosis patients in the Western Cape of South Africa [7]. The C strain is a drug-susceptible strain resistant to reactive nitrogen intermediates that spread among intravenous drug users [8]. The Haarlem strain belongs to a widely distributed genotype with reduced virulence in the murine model [9]. Genome-wide analyses of genetic variation among different MTB strains can help to unveil characteristics regarding the virulence and adaptation of this successful human pathogen.

In this study we analyzed the variation dynamics of the MTB genome by carrying out a multiple genome alignment of the six sequenced strains. The variations identified were then examined using a combinatorial approach that allows simultaneous comparison of all strains, rather than pair-wise comparisons of each to a reference genome, thus providing information about unexplored features of the MTB genome. This study gives insight into the evolution of the MTB genome and the mechanisms of adaptation of this pathogen to its human host.

## Methods

Multiple genome alignments were carried out with the freeware MAUVE v1.3 [10] using the genomes of MTB strains H37Rv (NC_000962), H37Ra (NC_009525), CDC1551 (NC_002755), F11 (NC_009565), C (NZ_AAKR00000000) and Haarlem (NZ_AASN00000000), the latter two from the Broad Institute  (See Additional File 1 for detailed Methods). A Perl-based package called *islandsanalyser *(Additional File 2) was designed to analyze the distribution and frequency of the LSPs identified in the alignment.

## Results and discussion

### Genome structure and genetic variation

The multiple genome alignment of the six MTB genomes showed extensive collinearity and the absence of widespread major rearrangement events. The limited number of rearrangements identified involved mobile genetic elements located at different sites along the genome. These variations were considered InDels, and not true rearrangements, when absent from some of the genomes in the dataset. This is exemplified by the phiRv1 phage, which was absent in the F11 and C strains and had a location that differed in strains H37Rv and H37Ra with respect to strains CDC1551 and Haarlem. Differential insertions of mobile genetic elements such as IS*6110 *were also detected and, again, considered as InDels when not present in the same location in all strains analyzed. This analysis indicated great stability in genomic structure and organization, characteristics that may be a consequence of the intracellular life-style of the tubercle bacilli, which limits the interaction with other microbial gene pools.

**Figure 1:**
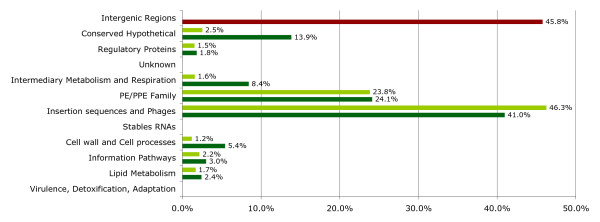
**Functional category classification of the LSPs found in the multiple alignments**. Dark green bars represent the variation in each category respect to the total of LSPs. The percentages are given with respect to a total of 166 matches because there are genes that are classified in more than one functional category. Light green bars represent the percentage of LSPs found respect to the total number of genes in each category. The percentage of the intergenic region category (red bar) represents the LSPs that involved a non-coding region.

Genetic variation in MTB is considered to occur through single nucleotide polymorphisms (SNPs) [11] and irreversible genetic events, such as large sequence polymorphisms (LSPs), which are believed to be the main source of phenotypic variability [12]. To determine the extent of genetic variation caused by LSPs, the polymorphisms identified in the multiple alignment were classified into different functional categories according to the genes affected (Figure 1). LSPs from 50 bp (the smallest size reported) to 12 kb were found to involve 120 genes. We did not find LSPs in genes for stable RNAs or in genes previously implicated in virulence, adaptation or detoxification. Two functional categories represented 65% of the total genes containing LSPs: 1) mobile genetic elements such as insertion sequences and phages and 2) the PE/PPE family of genes that contain repetitive sequences. This is consistent with the notion that these elements are known to drive the generation of small genetic polymorphisms [13]. The remaining LSPs were found in categories where it is common to find genes with redundant functions and suggests that MTB allows gene loss while avoiding deleterious effects. Finally, LSPs were detected in three genes annotated as transcriptional regulators (*Rv0823c, Rv0792c *and *Rv1353c*) that, although not essential for MTB survival [14], may be important in controlling gene expression under certain conditions.

The extent of LSPs present in intergenic regions had never before been determined for various genomes. We found that 45.8% of the total LSPs identified were present (complete or partially) in intergenic regions, indicating that variation is not targeted solely to coding sequences. This multiple alignment also identified LSPs in more genes, 120 versus 95, than would have been identified using microarrays to compare these same MTB strains against the H37Rv reference genome. This difference is due to the fact that our analysis was combinatorial and not limited to the identification of deletions with respect to a single reference genome. Thus this approach identified differential IS*6110 *integration sites, LSPs as small as 50 bp and LSPs in intergenic regions and genes of the PE/PPE family, which had not been investigated before [12]. These results, in addition to the SNP and microsatellite polymorphisms previously reported [15], indicate that the genetic variation within MTB is greater than previously expected.

### Distribution and frequency of polymorphisms

**Figure 2:**
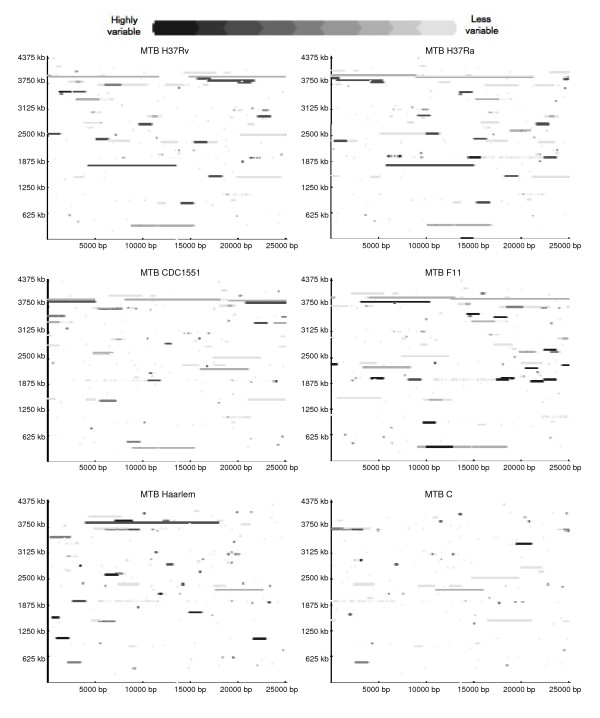
**Distribution and frequency of LSPs in strains H37Rv, H37Ra, CDC1551, F11, Haarlem and C**. The picture depicts LSP sites observed for each of the six strains (MTB H37Rv, H37Ra, CDC1551, F11, Haarlem and C) with respect to the other strains. The frequency of the LSP can be inferred from the color scale. Background color represents invariable positions. The base of the square is 25,000 bp and the sizes of the LSPs are proportionately scaled.

The observed skewed distribution of LSPs in the different functional categories could be the result of traits intrinsic to the MTB genome that constrain the accumulation of polymorphisms to specific regions of the chromosome. In this scenario, all the MTB strains should display similar patterns of LSP distribution. We therefore developed a tool (Additional file 2) to evaluate the presence and frequency of polymorphisms using a sliding window that identifies and represents variable regions as a continuous string, rather than as a step-wise count, for the presence of LSPs (as in a histogram). This avoids the over representation of small polymorphisms and the under representation of large polymorphisms that result from using discrete window presence/absence counts. The graphical output, which represents both LSP size and variability among strains, showed that the polymorphisms were spread across the entire genome and differed among strains, indicating that they were not constrained to the same genomic regions (Figure 2). As expected, highly variable regions corresponded to IS*6110 *insertion sites and the presence of the phiRv1 phage. This differential distribution and frequency of polymorphisms raised the possibility that there should be a limited number of polymorphisms unique to each strain.

**Figure 3:**
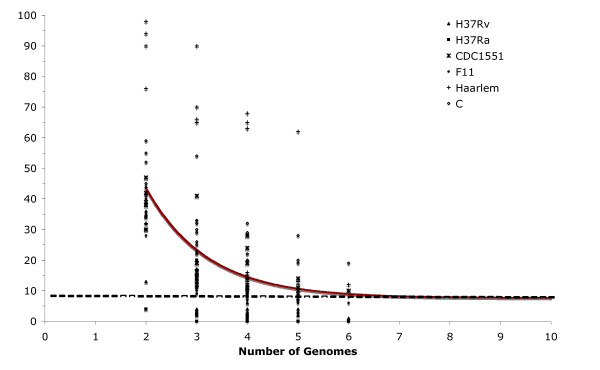
**Extrapolation of strain-specific deletions**. The number of specific deletions is plotted as a function of the number *n *of strains. For each *n*, markers are the 6!/[(*n *- 1)!·(6 - *n*)!] values obtained for the different strain combinations. The continuous curve represents the least-squares fit of the function *F_s_*(*n*) = κ_*s *_exp [-*n*/τ_*s*_] + Ω, where κ_*s *_= 186.7 ± 44.3, -1/τ_*s *_= -0.83 ± 0.13 and Ω = 7.6 ± 1.7. The best fit obtained had a correlation of 0.997.

**Figure 4:**
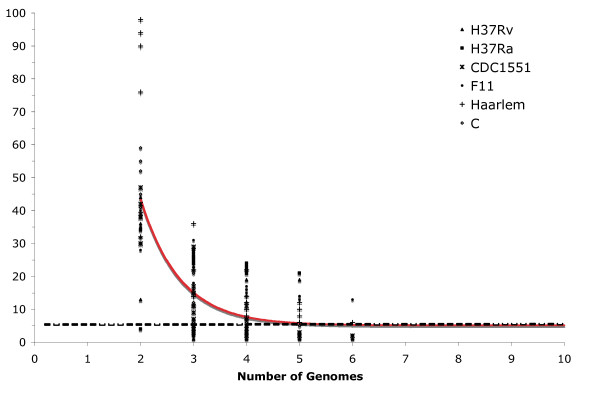
**Extrapolation of strain-specific insertions**. The number of specific insertions is plotted as a function of the number *n *of strains. For each *n*, markers are the 6!/[(*n *- 1)!·(6 - *n*)!] values obtained for the different strain combinations. The continuous curve represents the least-squares fit of the function *F_s_*(*n*) = κ_*s *_exp [-*n*/τ_*s*_] + Ω where κ_*s *_= 578.35 ± 198.07, -1/τ_*s *_= -1.36 ± 0.18 and Ω = 5.1 ± 1.1. The best fit was obtained with a correlation of 0.998.

To test this hypothesis we determined the number of strain-specific polymorphisms, independently of strain order, by the sequential addition and comparison of new MTB genomes. Strain-specific polymorphisms were separated into the following categories: *i) *specific deletions (SD), defined as a region absent in a particular strain and present in the rest of the dataset when *n*-1 genomes are compared, where n is the number of genomes compared; and *ii) *specific insertions (SI), a region unique to a particular strain and absent in the rest of the dataset when *n*-1 genomes are compared. The results obtained for the number of strain-specific deletions and insertions for all combinations of comparisons in a collection length of *n *= 2, 3, ...6, of the six genomes in the dataset were fitted to an exponential decay function to determine the number of specific deletion and specific insertions when the *n*th genome is included (Figures 3 and 4).

According to our hypothesis, the number of both types of strain-specific polymorphisms should decrease as more genomes are included. Interestingly, the value of strain-specific polymorphisms did not tend to an asymptotic value of zero but tended towards 7.6 ± 1.7 strain-specific deletions and 5.1 ± 1.1 strain-specific insertions when the *n*th genome is included. In addition, the variation dynamics differed among strains. For example, the laboratory strains presented fewer strain-specific polymorphisms, 1 SD and 1 SI in H37Rv and 2 SI in H37Ra, than clinical strains isolated during outbreaks. There were 12 strain-specific polymorphisms in CDC1551 (10 SD and 2 SI), 18 in Haarlem (12 SD and 6 SI) and 20 each in F11 (6 SD and 14 SI) and C (19 SD and 1 SI). This indicates that interaction with the human host is important in driving the generation and selection of genomic changes. The tendency observed by extrapolation of the number of genomes to infinite indicated that as additional genomes are included in the analysis only a small number of new strain-specific polymorphisms would be observed. The tendency to accumulate InDels also indicates that the MTB genome is under an active process of gene decay reminiscent of the adaptation process of obligate symbiotic bacterial pathogens [16].

### Genomic configuration of MTB strains

The genes involved in strain-specific deletions and insertions are summarized in Tables 1 and 2, respectively. Interestingly, we found that the avirulent laboratory strain H37Ra lacked specific deletions in comparison with the virulent strains, suggesting that loss of virulence in this strain is not a consequence of gene deletion. The larger genome of H37Ra, with respect to H37Rv, also suggests that virulent strains might enhance their fitness by means of gene loss. We also found that strain-specific deletions involving the PE/PPE gene family were identified only in clinical strains, consistent with their proposed function as variable surface antigens and mediators of interactions with host cells [17,18]. Other strain-specific deletions were also found in genes associated with the immune response. For instance, strain F11 lacked an entire gene encoding one of the culture filtrate proteins (CFP10A) known to mediate cellular immune responses [19]. The three LSPs related with transcriptional regulatory proteins correspond to strain-specific deletions of the Haarlem (*Rv0823c *and *Rv1353c*) and CDC1551 strains (*Rv0792c*). In terms of strain-specific insertions, unique insertion sites of the IS*6110 *element were predominant. Strain F11, for example, had 12 unique insertion sites for this element, suggesting that transposition events are important for variation in this particular genotype [20,21]. Both types of strain-specific polymorphisms were found in intergenic regions, with events such as insertion of transposons and duplication or loss of repeated regions between coding sequences. The identification and analysis of these intergenic regions is very valuable since they may be involved in regulation of adjacent genes or may contain as yet unknown small regulatory RNAs [22].

**Table 1:** Genes Involved in strain-specific deletions.

**Position**	**Size**	**Genes Involved**
		**H37Rv**

1987985	6830	MT1800 (Glycosyl transferase) – MT1801 (Molybdopterin oxidoredutase) – *mmpL14 *(Transmembrane transport protein)

		**CDC1551**

206981	58	Rv0175 (Probable conserved MCE Associated membrane protein)
888687	875	Rv0792c (GNTR-Family transcriptional regulatory protein) – Rv0793 (Hypothetical protein) – Rv0794c (Putative oxidoreductase)
2161299	139	*PPE34*
2349725	58	Intergenic Region between *pepE *(Xaa-Pro Dipeptidase) and Rv2090 (5'-3' Exonuclease)
2373345	58	C-terminal end of the Rv2112c (Hypothetical protein)
2382265	2275	*PPE37 *– *metH *(Methionine synthase)
2701639	501	Rv2406c (Conserved hypothetical protein) – Intergenic region – Rv2407 (Ribonuclease Z)
3727960	5284	*PPE54 *(Possible recombination event with *PE-PGRS49-50 *and *PPE55*)
3732032	192	PPE54
3948252	640	Rv3519 (Conserved hypothetical protein)

		**F11**

374076	61	PPE5
1509585	1101	Rv1334 (Conserved Hypothetical protein – Rv1335 (CFP10A) – *cysM *(cysteine synthetase)
1890753	172	Possible Promoter region of Rv1668c (Probable Macrolide ABC transport. ATP binding protein)
2381228	2645	*PE22 *and Intergenic region
3183106	109	Rv2859c (Hypothetical amidotransferase)
3702663	104	Intergenic region between *lpdA *(dihydrolipoamide dehydrogenase) and Rv3304 (Conserved hypothetical protein)

		**Haarlem**

912152	92	Rv0823c (Possible transcriptional regulatory Protein)
965426	481	*PE-PGRS15*
1451173	115	Intergenic region betwwen *thrB *(Homoserine kinase) and *thrC *(Threonine synthase)
1521604	4753	Rv1353c (Probable transcriptional regulatory protein) – Rv1354c (Possible diguanylate cyclase protein) – *moeY *(Molibdopterin biosynthesis protein) – Rv1356c (Hypothetical protein)
2060412	643	*PE-PGRS33*
2365930	1774	*helZ *(Helicase) – Rv2102 (Conserved hypothetical protein)
2546849	6480	Rv2271 (Hypothetical protein) – Rv2272 (Probable conserved transmembrane protein) – Rv2273 (Probable conserved transmembrane protein) – Rv2274c (Hypothetical protein) – Rv2275 (Hypothetical protein) – *cyp121 *(Cytochrome P450) – Rv2277c (Probable glycerolphosphodiesterase)
2792261	170	*PE-PGRS42*
3117314	439	Repeat region (DR) Intergenic between Rv2813 and Rv2814
3192481	87	IS*1539 *Transposase contained in repeat region
4033587	616	Rv3600c (Conserved hypothetical protein) – *panD *(Aspartate decarboxylase precursor)
4365135	84	*PPE69 *and Intergenic region with *PE36*

		**C**

334840	6611	*PE-PGRS3-PE-PGRS4-PPE3 *(partially absent in Haarlem Strain)
474110	125	Rv0401 (Probable conserved transmembrane protein)
565404	113	Intergenic region between Rv0480c (Probable amidohydrolase) and Rv0481c (Hypothetical protein)
956083	54	Intergenic region between *ercc3 *(Probable DNA helicase) and Rv0862c (Hypothetical protein)
1984522	109	C-terminal end *wag22 *(PE-PGRS)
2354142	214	*PE-PGRS36*
2456125	115	Intergenic region between *qcrB *(Ubiquinol cytochrome C reductase) and Rv2197c (Probable conserved transmembrane protein)
2825166	53	Rv2517c (Hypothetical protein)
2914707	115	*PE-PGRS44*
3113322	736	Repeat region DR Intergenic between Rv2816c and Rv2817c
3182209	54	Intergenic region between *frr *(ribosome releasing factor) and *pyrH *(Uridylate kinase)
3369008	113	*PPE47*
3369190	81	*PPE48*
3369344	62	*PE29*
3369422	171	*PE29*
3426378	83	Rv3074 (Hypothetical protein)
3911404	11522	*fadD17 *(Fatty-acid-CoA Synthase)*-PE-PGRS53-PE-PGRS54*
4009833	3486	*clpC1 *(Probable ATP-dependent protease) – *lsr2 *(Probable Iron-regulated protein) – *lysS *(lysyl t-RNA synthetase)
4143780	560	Rv3728 (Probable conserved two domain membrane protein – drug transporter)

Gene names are according to Tuberculist  and for genes absent in the MTB H37Rv genome the names are according to the MTB CDC1551 genome annotation [12]. Position indicated correspond to the site where the deletion has occurred.

**Table 2:** Genes Involved in strain-specific insertions.

**Position**	**Size**	**Genes Involved**
		**H37Rv**

3551227–3552586	1359	Intergenic region between Rv3185 (Probable transposase) and Rv3188 (Hypothetical protein)

		**H37Ra**

13627–14986	1359	Insertion of the IS*6110 *element between Rv0010c (Probable conserved membrane protein) and Rv0011c (Hypothetical protein)
1989343–1990797	1437	Insertion of the IS*6110 *element on the *ipl *locus

		**CDC1551**

804474–804691	217	Intergenic region between *rplW *(50S ribosomal protein L23) and *rplB *(50S ribosomal protein L2)
2341782–2341840	58	MT2144 (Hypothetical protein)

		**F11**

934662–936021	1359	IS*6110 *insertion in intergenic region between hypothetical proteins TBFG_10851 and TBFG_10853
1945992–1947351	1359	IS*6110 *insertion in Rv1724c (Hypothetical protein)
1992354–1993713	1359	IS*6110 *insertion in *cut1 *(Probable cutinase)
1997360–1998719	1359	IS*6110 *insertion in intergenic region between *wag22 *(PE-PGRS) and Rv1760 (Hypothetical protein)
2004182–2005541	1359	IS*6110 *insertion in intergenic region between hypothetical proteins Rv1765c and Rv1766
2017862–2019221	1359	IS*6110 *insertion in *cyp144 *(Cytochome P450)
2270442–2271801	1359	IS*6110 *insertion in Rv2015c (Hypothetical protein)
2349289–2350648	1359	Intergenic region between Rv2077c (Possible conserved transmembrane protein) – Rv2078 (Hypothetical protein)
2697325–2698684	1359	IS*6110 *insertion in Rv2390c (Hypothetical protein)
3492136–3493495	1359	IS*6110 *insertion in Rv3113 (Probable phosphatase)
3564263–3565622	1359	IS*6110 *insertion in intergenic region between Rv3183 (Possible transcriptional regulatory protein) – TBFG_13208 (Conserved hypothetical protein)
3831596–3831772	176	Intergenic region between Rv3401 (Hypothetical protein) and Rv3402c (Hypothetical protein)
3853062–3860408	2394	TBFG_13461 (Hypothetical protein) – TBFG_13462 (Hypothetical protein) – TBFG_13463 (Hypothetical protein) – TBFG_134614 (Probable Transposase) – Intergenic region

		**Haarlem**

1071617–1072976	1359	IS*6110 *insertion in Rv0963c (Conserved hypothetical protein)
1714841–1716200	1359	IS*6110 *insertion in *mmpl12 *(Transmembrane protein)
1834511–1834569	58	Rv1637c (Hypothetical protein)
1973210–1973436	226	PPE24
2606037–2607396	1359	IS*6110 *insertion in Rv2336 (Hypothetical protein)
4144939–4144997	58	*leuA *(2-isopropylmalate synthase)

		**C**

4088473–4088705	232	Rv3680 (Probable anion transporter ATPase)

Gene names are according to Tuberculist when homologous to MTB H37Rv, if not, genes are named according to the annotation of the respective genome.

Strain-specific polymorphisms may also help to explain some of the observed phenotypic differences among MTB strains. This study is therefore hypothesis-generating and functional analyses of the polymorphisms found can be carried out to understand how genetic variation affects the outcome of infection. Our results indicate that circulating strains harbor a higher number of strain-specific polymorphisms (Tables 1 and 2) and suggest that interaction with the human host results in strain-specific genomic polymorphisms that may in turn determine the phenotypic differences among circulating MTB strains. While most deletions tend to be slightly deleterious [12], some strain-specific polymorphisms, such as the Beijing genotype's strain-specific deletion in the *pks1/15 *region [4,23], may contribute to the pathogen's virulence or its adaptation and the establishment of stable associations with the human host population [24]. The tendency predicted here regarding genetic variation of the MTB genome is based on six strains that represent only a fraction of the global MTB population. Thus it is necessary to further investigate the extent of intra and inter lineage genetic variation for a more accurate prediction of the variation dynamics within the entire MTB population. The inclusion of a larger number of representative strains can provide a broader understanding of how these genetic polymorphisms contribute to the adaptation of certain genotypes to human populations.

## Conclusion

The multiple alignment of six MTB genomes identified both frequency and differential distribution patterns of polymorphisms. This study indicated that circulating MTB strains are under active selective pressure that leads to variation by means of gene loss or inactivation and suggests that this species in a process of gene decay. The identification of strain-specific polymorphisms may ultimately translate into molecular markers for strain surveillance and predictors of outbreaks in defined human populations.

## Abbreviations

MTB: *Mycobacterium tuberculosis; *LSP: Large sequence polymorphism; InDels: Insertions and Deletions; SNP: Single nucleotide polymorphism; SD: Specific Deletion; SI: Specific Insertion

## Authors' contributions

ACR carried out the multiple alignments and genome comparisons, designed the *islandsanalyser*, evaluated data, analyzed the results obtained and was in charge of writing the manuscript. MMZ supervised the progress of the work, helped in writing the manuscript and contributed with discussions regarding the overall research and the results obtained. JM carried out the programming of the *islandsanalyser*.

## Supplementary Material

Additional file 1 **Methods.** Click here for file

Additional file 2 ***Islandanalyser *****package.** The scripts were built using Perl 5. In order to execute them the module Chart::Plot (available at ) and the module GD  must be installed. It works on any operating system that supports Perl, a list of systems where Perl is available can be obtained at . Contents: genoma.pl, max.pl and Graph.pl and proper documentation. Click here for file
