# Supplementary material for: Analysis of the genetic variation in Mycobacterium tuberculosis strains by multiple genome alignments
Source: BMC Res Notes. 2008 Nov 7;1:110. doi: 10.1186/1756-0500-1-110 (PMC2590607; doi:10.1186/1756-0500-1-110)
Supplement: Additional file 1 — Methods. [file 1756-0500-1-110-S1.doc]

### Additional file 1 – Materials and Methods

**Methods**

### Genomic sequences

The accession numbers of the genomic sequences of the six MTB strains are: H37Rv (NC_000962), H37Ra (NC_009525), CDC1551 (NC_002755), F11 (NC_009565), Haarlem (NZ_AASN00000000) and C (NZ_AAKR00000000). Detailed information about the genome projects of the F11, C and Haarlem strains can be found at the Broad Institute web site [www.broad.mit.edu](http://www.broad.mit.edu/) [1]

### Multiple genome alignments

Multiple genome alignments were carried out with the freeware MAUVE v1.3 [2] using the following default parameters: Seed size, Determine LCBs, Extend LCBs and Full Alignment options were enabled; the value for Minimum island size, Maximum backbone gap size and Minimum backbone size was 50 and the aligner selected was ClustalW 1.8.4. The genomes of strains H37Rv, H37Ra, CDC1551 and F11 were loaded as Genbank files; the supercontigs of the unfinished sequences of the C and Haarlem strains were loaded as Fasta files ordered with respect to the genomes of the fully assembled strains. The genetic variation was studied using MAUVE’s output files (*.islands*) that contains tab-delimited text listing the exact coordinates of genomic islands found in the alignment. Each island represents a region of the alignment where one or more genomes have a LSP element absent in one or more of the other genomes [2]. The *.islands* file of this alignment was edited manually in order to eliminate islands corresponding to undetermined draft regions in the genomes of the Haarlem and C strains. These LSP genomic locations derived from the multiple comparison were then scrutinized by evaluating the presence or absence of islands in each genome when all possible combinations were performed. This novel combinatorial approach was used to mine for genetic variation in the strains analyzed.

### LSP distribution and frequency

A Perl-based package called *islandsanalyser* (Additional File 2) was designed for the visualization of the distribution and frequency of the LSPs identified in the alignment. The script reads the .*islands* file and creates a presence/absence matrix of a length equal to the size of the genome, where every nucleotide position of each genome compared is assigned a value depending on its degree of conservation in the other genomes. The matrix starts with a value of 0 for every nucleotide position. If a region was identified in the alignment as a LSP, all the nucleotides within this region acquired a value of 1, if the polymorphism occurred in more than one genome the value of these nucleotides rose progressively to a maximum value of (*n*-1), where *n* is the number of genomes being compared. In our comparison of six genomes, the nucleotides contained in a region that was present only in one genome were assigned a value of 5, whereas those found in a region conserved in all genomes were given a value of 0. Once the matrix was filled, the variability at each nucleotide position was analyzed by using a sliding window of size *w* that incorporates the sum of the values contained in the (*w*-1)/2 adjacent positions, thus ensuring that the value is assigned to the nucleotide located in the middle position of the window. The result of this step was a new matrix where the variability of each nucleotide position is measured taking into account the degree of variation of the nucleotides in its vicinity. Each nucleotide position was then plotted on a square-based graph to represent the length of the genome using a base of 25,000 bp. The value of each nucleotide position was converted into a color scale where the highest value (most variable) is represented in black and the lowest is represented in light grey; invariable nucleotides were not plotted and conserved the background color. The window size *w* used in the evaluation of the genomes was 50 as it was the smallest size reported in the alignment.

### Functional category classification

The .*islands* file was processed to eliminate the LSPs that shared the same genome coordinates (those that had happened in more than one genome), leaving only one representative LSP and thus ensuring no redundancy in the genes classified. It was assumed that the majority of the LSPs identified in the alignment corresponded to deletion events so the sequences still present in other MTB strain were used to classify the functional category of those genes within polymorphisms. For IS*6110* insertion events, we only considered unique transposition events (those present only in different locations). Sequences of all the different LSPs were extracted from each corresponding genome and were compared by BLAST with the genes present in each of the eleven functional categories described for the MTB genome [3]. For the classification of LSPs that involved intergenic regions, the comparison was performed using as database the genomic regions found between every predicted open reading frame on the MTB H37Rv genome [3].

### Extrapolation of strain-specific polymorphisms

The information of the .*islands* file was used to determine the number of specific polymorphisms (separated in specific deletions and specific insertions) of each strain when the sequential inclusion of up to six strains was simulated in all possible combinations. The method for determining the core genome of a bacterial species [4] was adapted for the extrapolation of strain-specific polymorphisms. By measuring the number of specific polymorphisms as a function of the number of sequenced strains, it is possible to extrapolate the number of specific polymorphisms when the *n*th genome is sequenced. For *n*=1, 2, … 6, the number *N* of independent measurements of specific polymorphisms present in the *n*th genome is: *N* = 6!/[(*n* – 1)!·(6 – *n*)!]. This formula gives the number of points reported in Figures 3 and 4 as a function of *n*. The number of strain-specific polymorphisms for a large number of sequenced strains was extrapolated by fitting the exponential decay function *Fs*(*n*) = κ*s* exp [−*n/*τ*s*] + Ω to the amount of specific polymorphisms identified in a total of 186 comparisons. In the equation, κ*s* is the amplitude of the exponential decay, τ*s* is the decay constant that measures the speed at which *Fs*(*n*) converges to its asymptotic value, and Ω measures the number of strain-specific polymorphisms for *n* → ∞ , where *n* is the continuous extrapolation of the number of strains taken into account [4]. Results of the fitting procedure were evaluated with Pearson’s correlation coefficient.

### References
