# Supplementary material for: Analysis of the genetic variation in Mycobacterium tuberculosis strains by multiple genome alignments
Source: BMC Res Notes. 2008 Nov 7;1:110. doi: 10.1186/1756-0500-1-110 (PMC2590607; doi:10.1186/1756-0500-1-110)
Supplement: Additional file 2 — Islandanalyser package. The scripts were built using Perl 5. In order to execute them the module Chart::Plot (available at ) and the module GD must be installed. It works on any operating system that supports Perl, a list of systems where Perl is available can be obtained at . Contents: genoma.pl, max.pl and Graph.pl and proper documentation. [file 1756-0500-1-110-S2.zip › Islandsanalyzer/html/genoma.html]

xml version="1.0" ?


genoma - Analyze island files and create a TSV description of their window averaged differences.


- NAME
- SYNOPSIS
- DESCRIPTION


---

# NAME

genoma - Analyze island files and create a TSV description of their window averaged differences.

---

# SYNOPSIS

```
 genoma.pl -f <island-file> -c <genome-count> -w <window-size>
```

```
 -f      : The name of the island file
 -c      : The number of genomes to analyze
 -w      : Window size to use
 -o      : File to write the output to. Default output.tsv
```

---

# DESCRIPTION

This program is meant to perform the first step in converting an island file into a graph.

Genoma performs the following steps:

1. Read the island plain text file into a memory structure. The memory structure is simply a matrix in $genomes[$a][$b] where $a is the number of the genome and $b is the position within that genome.
2. With this matrix of 0 and 1 and a window of size $w (provided by the user on the script's startup arguments) the system put into each position ($col) the sum of the $w sized chunk of genome pieces. By doing this the position $genome[$a] contains the sum of the elements between $genome[$a-$w/2] and $genome[$a+$w/2].
3. This genome information that has just been created is now written to tab separated file, with one line for each genome, and inside each line the list of window added values for each position.
