# Supplementary material for: Analysis of the genetic variation in Mycobacterium tuberculosis strains by multiple genome alignments
Source: BMC Res Notes. 2008 Nov 7;1:110. doi: 10.1186/1756-0500-1-110 (PMC2590607; doi:10.1186/1756-0500-1-110)
Supplement: Additional file 2 — Islandanalyser package. The scripts were built using Perl 5. In order to execute them the module Chart::Plot (available at ) and the module GD must be installed. It works on any operating system that supports Perl, a list of systems where Perl is available can be obtained at . Contents: genoma.pl, max.pl and Graph.pl and proper documentation. [file 1756-0500-1-110-S2.zip › Islandsanalyzer/html/graph.html]

xml version="1.0" ?


graph - Take a TSV description of window averaged differences for island files and create a 2D graph of it.


- NAME
- SYNOPSIS
- DESCRIPTION


---

# NAME

graph - Take a TSV description of window averaged differences for island files and create a 2D graph of it.

---

# SYNOPSIS

```
 graph.pl -g <genome-number> <options> < <genome-result-file> > <png-output-file>
```

```
 -g : The number of the genome to analyze. 1 is the first
 -n : Set the minimum y to use (0)
 -x : Set the maximum y to use (unbounded)
 -a : Set the minimum x to use (0)
 -b : Set the maximum x to use (unbounded)
```

---

# DESCRIPTION

This program is meant to perform the second step in converting an island file into a graph.
The matrix in the entry is supposed to be sparse, that is, it is supposed to contain a lot of zeroes. This program plots the places that contain numbers different from zero as colored spaces on a graph. Because the entry data was ``window normalized'' colores spaces will tend to cluster.

With this in mind a point in the graph has three attributes:

- The X position which gives the position of the difference within the genome in intervals of 25000. This means that if the point is in position 10 in X it will correspond to position 25000 \* 10 in the actual genome.
- The Y position which gives the position of the difference within the genome as a modulo of 25000. This means that point that have the same Y but different X are not really related.
- The color of the point, the darker the more differences there are in the selected spot. By default the program is configured to use 255 as the biggest possible value for the sum of differences. If the maximum amount is more than 255 then points with values of 255 and more will all be black, thus making it impossible to distinguis within them. On the other hand, is the maximum amount is less that 255 then the graph will have no significantly dark areas, even if there are differences.

This script uses the Chart::Plot to create the graph and writes to standard output as a PNG file.
